# Obstructive Uropathy Secondary to Accidental Ureteric Placement of an Indwelling Urinary Catheter at the Time of Caesarean Section: A Case Report

**DOI:** 10.7759/cureus.80674

**Published:** 2025-03-16

**Authors:** Sara E Hariz, Priti Pradhan

**Affiliations:** 1 Obstetrics and Gynaecology, Women's and Children's Hospital, Adelaide, AUS

**Keywords:** caesarean, iatrogenic ureteric injury, lower segment caesarean section, obstructive uropathy, urinary catheter

## Abstract

This case describes a rare complication of Foley catheter insertion where accidental instrumentation of a ureter occurred at the time of caesarean section with the subsequent development of bladder obstruction and acute kidney injury. Rapid identification and management can minimise the associated long-term implications; this therefore necessitates greater awareness of this possible complication. The current understanding of this condition recognises neurogenic bladder and female sex as risk factors; however, physiological changes in pregnancy may also act as a significant risk factor for this complication.

## Introduction

Foley catheter insertion is a routine practice both at the time of caesarean section and in labour for certain indications. We describe a case where a catheter was inserted at the time of caesarean section, where it was accidentally placed and inflated in the left distal ureter, a complication not discovered until the following day. On review of the literature, there is a potential of increased risk of misplacement into the ureters in the context of female sex and neurological conditions such as neurogenic bladder, spinal cord injury, stroke, dementia, myasthenia gravis or multiple sclerosis [1,2]. However, multiple cases have been described in pregnant or immediately post-partum patients [3-7], which may suggest that the gravid uterus alters the position of the uretero-vesical orifices in conjunction with the physiologic hydronephrosis associated with pregnancy, allowing for an increased risk of accidental instrumentation of the ureter. We also explore methods of identifying potential aberrant catheter placement to allow for earlier diagnosis, which is particularly important in this population, which often has altered sensation in the context of epidural or spinal analgesia.

This article was previously posted to the Research Square preprint server on April 18, 2024.

## Case presentation

**Figure 1:**
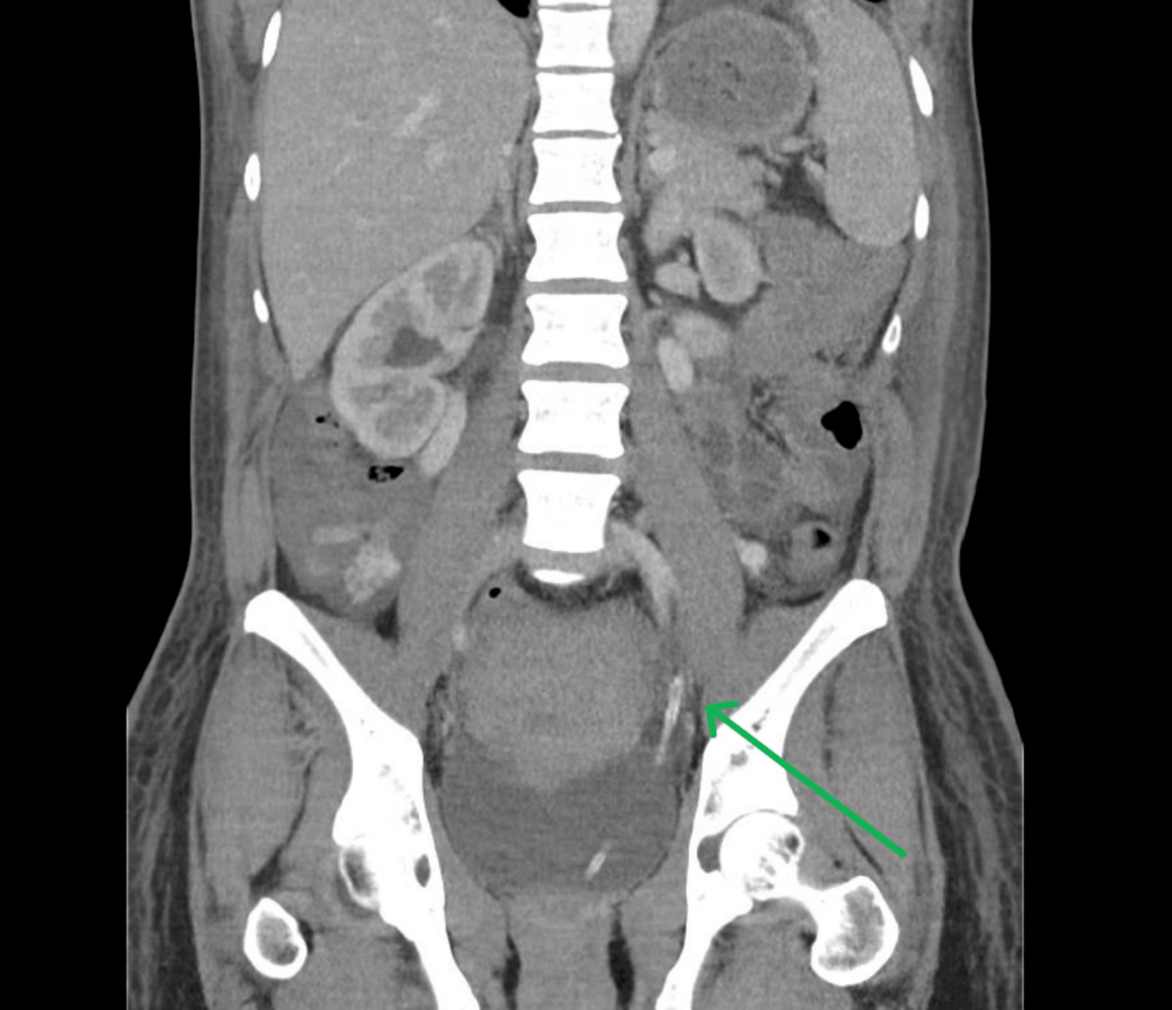
Computed tomography (CT) coronal

**Figure 2:**
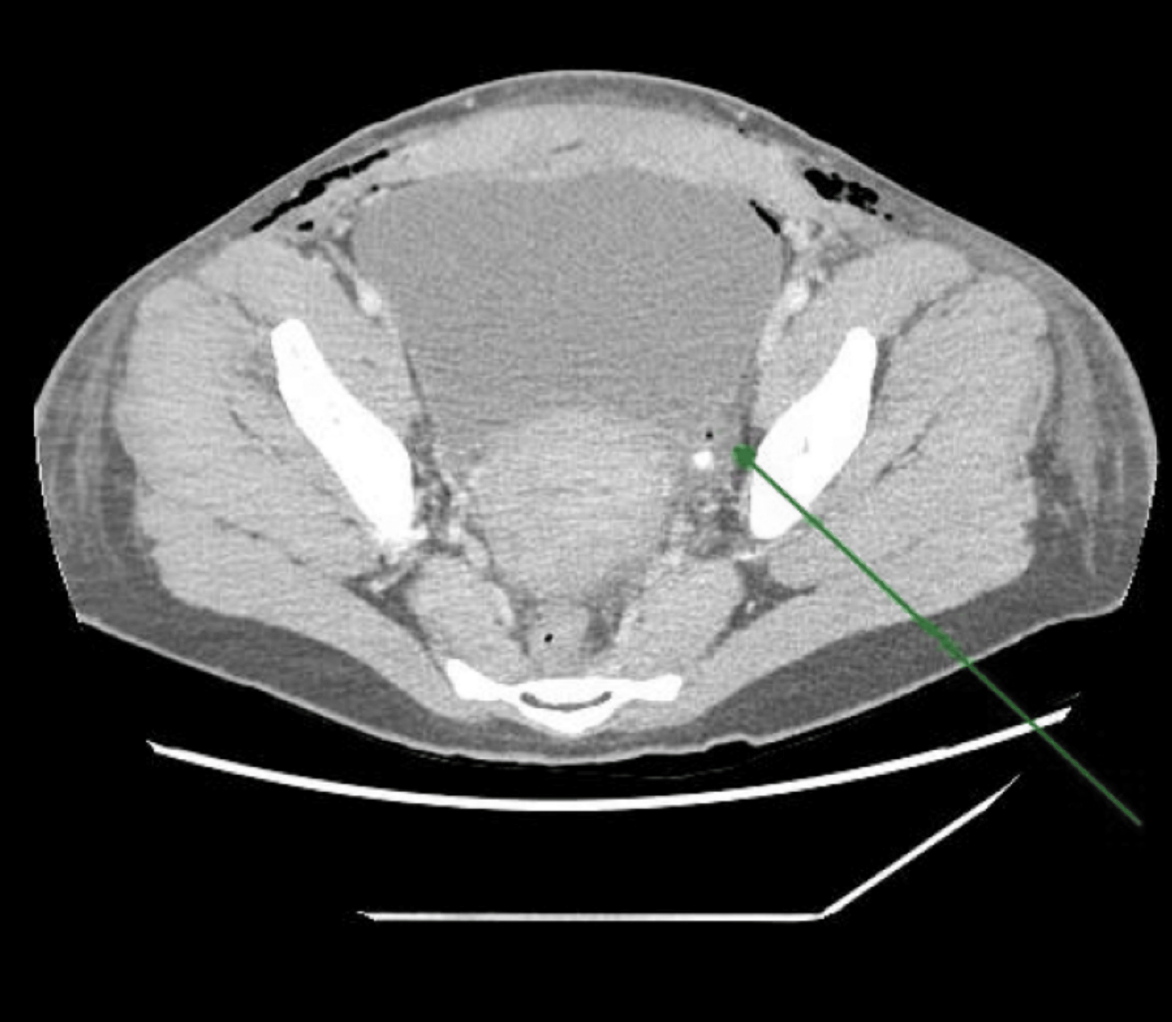
Computed tomography (CT) transverse

A 31-year-old female underwent an uncomplicated lower segment caesarean section (LSCS) at term for dichorionic diamniotic (DCDA) twins where an indwelling catheter (IDC) was placed after the administration of spinal anaesthesia for an elective LSCS. The following morning, a medical review was requested for a urine output of 0.2 ml/kg/hr of dark, concentrated urine, which had not improved overnight despite significant fluid resuscitation. Biochemical analysis showed a significant acute kidney injury with a creatinine of 120 umol/L and urea of 6.0 mmol/L (Table 1). Throughout the proceeding hour, she developed significant abdominal pain and CT confirmed that the IDC tip was protruding 5 cm into the left ureter with bilateral hydronephrosis (Figures 1-2). Retraction of the catheter resulted in decompression of the bladder and resolution of the obstructive uropathy. Subsequent follow-up ultrasound confirmed no evidence of long-term complications.

**Table 1:** Biochemistry

Biochemistry	Reference ranges	Baseline	One Day Post Injury	Two Days Post Injury	Three Days Post Injury	Four Days Post Injury
Creatinine	30-70 umol/L	54	120	106	79	66
Urea	1.2-4.0 mmol/L	3.2	6.0	6.4	4.3	2.7

## Discussion

This is a notably rare complication of urinary catheter placement; however, of the small number of known cases, the identified risk factors were female sex and neurogenic bladder [2]. On review of the literature, we have found five other cases documented in pregnant or immediately postpartum patients [3-7]. Physiological changes in pelvic anatomy in pregnancy such as hydronephrosis may contribute to the risk of this complication [3,5]. Of these cases, the majority of pregnancies involved the right ureter rather than the left. Dilation of the right ureter in pregnancy is a known phenomenon likely contributed to by the dextrorotation of the uterus and the location of the right ureter crossing over the pelvic brim [8,9]. This may explain the higher incidence of accidental instrumentation, however, interestingly, the contralateral side was instrumented in this case. Furthermore, females are deemed at higher risk due to a shorter urethra [10]. The gravid uterus in the setting of twin pregnancy may have caused protrusion of the ureteric orifices in line with the urethral orifice, thus leading to accidental instrumentation. Proposed methods of identification include recognition of the increased length of the catheter inside the patient, reduced urine output non-responsive to fluid resuscitation, and acute kidney injury [3,10]. Our patient also had pain secondary to bladder distension despite a draining catheter. A significant proportion of patients also developed pyelonephritis; however, of this group, none were pregnant or postpartum, presumably due to the need for longer-term catheterisation to predispose them to this complication than typically seen in the postoperative or postpartum population [8]. Clinician experience and safety protocols regarding the distance of insertion, not inserting to the hilt in females and ensuring adequate urine drainage prior to inflation are possible mechanisms of reducing the incidence of this complication [2]. Whilst this is an exceedingly rare complication, practitioners should consider this in addition to other causes in patients who are oliguric post caesarean section and further investigate with a thorough examination, biochemical analysis and imaging, if indicated.

## Conclusions

This case underscores the importance of vigilance during catheter placement, particularly in the context of caesarean sections due to the anatomical shift to pelvic organs caused by a gravid uterus. Although an exceedingly rare complication, obstructive uropathy due to ureteric catheter misplacement demands consideration in the differential diagnosis of postoperative oliguria. Heightened awareness among practitioners can facilitate early identification, prompt intervention, and prevention of potential long-term complications.
